# Clinical characteristics and associated factors of constipation patients with type 2 diabetes

**DOI:** 10.3389/fendo.2026.1698701

**Published:** 2026-02-16

**Authors:** Yan Fang, Jiahao Chen, Chendong Ni, Yi Dai, Lili Cai, Xiaohua Yang, Ji Hu, Hong-Hong Zhang

**Affiliations:** 1 Department of Endocrinology, the Second Affiliated Hospital of Soochow University, Suzhou, China; 2 Department of Endocrinology, PuTuo District Liqun Hospital, Shanghai, China; 3 Department of Endocrinology, the Affiliated Haian Hospital, Nantong University, Nantong, China

**Keywords:** CGM, coefficient of variation, constipation, glucose target time in range, T2DM

## Abstract

**Aim:**

The aim of this study was to explore the clinical characteristics and associated factors of constipation in patients with type 2 diabetes (T2DM). Here, we focus on the correlation between time in range (TIR) and coefficient of variation (CV) of glucose and constipation in patients with T2DM.

**Methods:**

In the exploratory, cross-sectional study, a total of 120 patients diagnosed with T2DM in the department of Endocrinology and Gastroenterology of Liqun hospital from 2023 to 2024 were recruited. Patients with concurrent constipation were included in the constipation group, and those without concurrent constipation were included in the non-constipation group. Fasting blood indicators of patients were detected, including fasting blood glucose (FBG), 2 hours postgrandial blood glucose (2hPBG), glycosylated hemoglobin type A1C (HbA1c), fasting C-peptide (FCP), homeostasismodel assessment-insulin resistance (HOMA-IR), triglyceride (TG), total cholesterol (TC), high density lipoprotein cholesterol (HDL-C), and low-density lipoprotein cholesterol (LDL-C). Blood Glucose was monitored by a silicon-based dynamic system for 14 days, and TIR and CV were calculated by a continuous glucose monitoring system (CGM). In addition, the symptoms of constipation, gastroparesis cardinal symptom index (GCSI), patient assessment of upper gastrointestinal symptom severity index (PAGI-SYM) and patient assessment of quality-of-life index (PAGI-QOL) were evaluated by questionnaires. The impact of TIR and CV for constipation risk was evaluated using receiver operating characteristic (ROC) curves.

**Results:**

1. More serious condition in constipation symptoms, GCSI, VSI, PAGI-SYM and PAGI-QOL scores in the constipation group. 2. Compared with the non-constipation group, the constipation group had longer courses, and higher probabilites of complications of autonomic neuropathy, diabetic nephropathy and hyperlipidemia; 3. Compared with the non-constipated group, FCP was significantly decreased, and HbA1c, TC and LDL-C were significantly increased in the constipation group. 4. TIR and CV were significantly correlated with constipation in patients with T2DM.

**Conclusions:**

Lower TIR and higher CV were associated with constipation in T2DM patients. These findings suggest that CGM-derived metrics may be useful markers of constipation risk and warrant investigation in prospective studies to determine whether improving these parameters can prevent constipation development.

## 1 Introduction

Diabetes mellitus is a chronic metabolic disease characterized by hyperglycemia and varying degrees of insulin deficiency and/or insulin resistance. Type 2 diabetes mellitus (T2DM) is more common in adults, accounting for more than 90% of the diabetic population. Patients with T2DM have an increased risk of various gastrointestinal complications, including gastroesophageal reflux, esophageal peristalsis disorders, impaired gastric relaxation and regulation, impaired gastric contraction and emptying, dyskinesia of intestinal tract, and non-alcoholic fatty liver disease (1). Studies have shown that about 75% of patients with T2DM suffer from gastrointestinal symptoms, such as heartburn, acid reflux, non-cardiac chest pain, dysphagia, postprandial satiation with nausea, abdominal distention, abdominal pain, diarrhea, constipation and fecal incontinence (2, 3). Diabetic gastrointestinal diseases significantly delay the body’s digestion and absorption of food, causing patients to have gastrointestinal discomfort and reducing the quality of life of patients. Up to 50% of patients with diabetic gastrointestinal diseases have symptoms of anxiety or depression, which affects the mental state of patients (4). Among them, studies have reported a prevalence of T2DM with constipation ranging from 11% to 56% (5, 6). The underlying causes of constipation in diabetic patients may be multifactorial and strongly associated with the increased risk of autonomic and intestinal neuropathy. In one study, diabetic patients with autonomic dysfunction were more likely to develop constipation than diabetic patients without autonomic dysfunction (22% vs 9%) (7, 8).

Hemoglobin A1c (HbA1c) level has long been considered the gold standard for evaluating the quality of glycemic control, and thus the overall quality of diabetes management. However, studies have found that HbA1c has limitations, such as the lack of quantification of blood glucose fluctuations and the actual number of hypoglycemic episodes. The need for implementing new effective blood glucose control indicators has increased, and Continuous Glucose Monitoring Systems (CGMS) came into being (9). Glycemic Variability (GV) refers to the fluctuation of blood glucose levels, which indicates the amplitude of blood glucose fluctuations in a patient during a certain time interval. Currently, GV becomes a valuable tool for assessing diabetes management. The American Diabetes Association (ADA) established thresholds for the main metrics to quantify GV and described their application in clinical practice (10). Based on these data, standard reports are generated, which are visual records of blood glucose values and contain the main CGM indicators, such as the mean value of blood glucose (MBG), Time in Range (TIR), and Coefficient of Variability (CV) (10, 11). TIR itself is not an indicator of GV, but can indirectly provide valuable information about the quality of glycemic control and the extent of GV. Current guidelines have emphasized the role of TIR in clinical practice and pointed out its advantages over HbA1c (12). CV is obtained by dividing the standard deviation by the MBG, and is the GV index adjusted according to MBG (13).

Recently, there has been increasing interest in assessing the correlation of CGM, such as TIR and CV, with the development of chronic diabetic complications, including diabetic peripheral neuropathy, diabetic gastroparesis, diabetic retinopathy and diabetic foot (14, 15). However, studies on the effects of TIR and CV on T2DM with constipation are rare. So, we conducted the exploratory, cross-sectional study. The present study will fill this gap and has significance to the prevention and treatment of diabetes complicated with constipation.

## 2 Methods

### 2.1 Participants

According to the diagnostic criteria of T2DM and constipation, 120 patients treated in the Department of Endocrinology and Gastroenterology of Liqun Hospital of Putuo District in Shanghai from 2023–2024 were enrolled, including 60 patients with constipation (Constipation group) and 60 patients with non-constipation (Non-constipation group). The sample size was calculated using online software (http://shinystats.org/). Inclusion criteria:(1)All patients met the diagnostic criteria of T2DM in the Chinese Guidelines for the Prevention and Treatment of T2DM (2020 edition).(2)Constipation was diagnosed according to Rome IV functional constipation criteria.(3)The age is between 18 and 80 years old, and gender is not limited.(4)No recent use of drugs affecting defecation.(5)Consciousness is clear, mental normal, and cooperate with relevant treatment.(6)Sign the informed consent form. Exclusion criteria:(1)History of chronic constipation before T2DM.(2)Organic constipation caused by systemic diseases.(3)Abnormal function of heart, lung, kidney and other organs.(4)Complicated with other acute and chronic reactive diseases or other uncontrollable basic diseases.(5)Cognitive dysfunction or consciousness disorders, unable to cooperate;(6) Incomplete medical history and withdrawal from the study.

### 2.2 Questionnaire and scoring criteria

(1) Gastroparesis Cardinal Symptom Index (GCSI). GCSI is composed of three sub-scales: nausea/vomiting sub-scale, postprandial bloating/early saturated sub-scale, and bloating sub-scale, which are used to assess the severity of gastrointestinal symptoms in patients. The nausea/vomiting sub-scale is composed of the following 3 items: nausea, retching, vomiting. The postprandial fullness/early satiety sub-scale consists of the following 4 items: bloating (feeling full), decreased food intake, postprandial fullness, and decreased appetite. The bloating subscale consists of 2 items: bloating and satiety (distention of the abdomen). GCSI is composed of 9 items, and each item is scored on a 6-point scale according to the severity of the patient’s symptoms, including no symptoms (0 points), very mild (1 point), mild (2 points), moderate (3 points), severe (4 points) and very severe (5 points). The sub-scale score refers to the average score of each item in each sub-scale, and the total GCSI score is the average score of the three sub-scales. Patients with GCSI score ≥1.9 are considered to have gastroparesis symptoms, and the higher the score, the more severe the symptoms are.
(2) Intestinal sensitivity index (VSI). VSI score describes a person’s response to symptoms of abdominal discomfort, including abdominal pain, diarrhea, constipation, difficulty swallowing, and a sense of urgency. The scoring scale has 15 questions, and the responses are divided into 6 levels, including very consistent (1 point), relatively consistent (2 points), consistent (3 points), inconsistent (4 points), relatively inconsistent (5 points), and very inconsistent (6 points). The evaluation total score is the sum of the scores of all questions.
(3) Patient Assessment of Upper Gastrointestinal Symptom Severity Index (PAGI-SYM score). PAGI-SYM score is a classification of clinical manifestations and severity of gastrointestinal diseases. The symptoms that have occurred in the past 2 weeks are recorded and the severity is scored. Score reference: 0: asymptomatic; 1: very slight; 2: light; 3: moderate; 4: heavy; 5: Extremely severe. The evaluation total score is the sum of the scores of all questions.
(4) Patient Assessment of Quality of Life Index (PAGI-QOL score). PAGI-QOL score is about the impact of gastrointestinal diseases on the quality of life and happiness of patients. Rating references: 0: never; 1: Occasionally; 2: sometimes; 3: Often; 4: Most of the time; 5: Always. The evaluation total score is the sum of the scores of all questions.
(5) Constipation symptoms. Patients were scored according to frequency of defecation, stool characteristics (according to the Bristol stool chart), duration of defecation, bloating and how they felt during defecation. The total score is the sum of all the scores.

The above diagnostic questionnaire should be filled in by oneself as far as possible. If the patient is illiterate, the investigator can orally ask questions according to the questionnaire content and fill in it for the patient. If the patient does not understand the survey content clearly, the investigator should explain it many times to make the patient understand. The detailed information of the above questionnaire is shown in the appendix.

### 2.3 Clinical and biochemical measurements

Basic data of all the patients were collected, including gender, age, and duration of diabetes. Body mass index (BMI) was calculated according to the formula: BMI (kg/m²) = weight(kg)/height(m)². Diabetes complications were also recorded. All patients fasted for at least 8 hours before blood samples were collected. The fully automated blood cell analyzer (Sysmex XN-2000, Japan) was used to determine blood routines. Biochemical parameters, such as fasting blood glucose (FBG), 2 hours postgrandial blood glucose(2hPBG), triglyceride (TG), total cholesterol (TC), high-density lipoprotein cholesterol (HDL-C), low-density lipoprotein cholesterol (LDL-C) were measured with an automated biochemical instrument (AU5800). Fasting C-peptides (FCP) was measured with a fully automated chemiluminescence immunoassay analyzer (MAGLUMI X8). Glycated hemoglobin A1c (HbA1c) was measured with a TOSOH HLC-723 G8. The function of β cell was calculated using modified Homa formula: Homa-IR=0.27×FCP/(FBG-3.5). A silicon-based dynamic blood glucose monitoring system was used to monitor blood glucose continuously for 14 days, and TIR and CV were calculated by system formulas.

### 2.4 Statistical methods

Software SPSS 26.0 was used for data analysis. All data were expressed as means ± standard deviation. Normality was checked for all data before analysis. Comparisons between both groups were tested using two-sample *t*-test, Mann-Whitney *U* test, or *χ*2 test. Multiple logistic regressions analysis with stepwise method was used to evaluate the influencing factors of constipation in patients with T2DM. The impact of the constipation risk factors was compared using the Area Under the Curve (AUC) of the Receiver Operating Characteristic (ROC). The accuracy was assessed by the sensitivity and specificity of the risk factors. A *p* value less than 0.05 was considered to be statistically significant.

## 3 Results

### 3.1 Constipation symptom scores of the two groups

The constipation symptom scores of patients in the diabetes with constipation group and the non-constipation group are shown in **Table 1**. It showed that the scores of diabetes with constipation were mostly concentrated in the 1–3 points, while the scores of non-constipated patients were mostly concentrated in the 0–1 points.

**Table 1:** Comparison of constipation symptom score between the diabetic patients with constipation and without constipation.

Symptom Domain	0 point		1 points		2 points		3 points	
	Non- constipation	Constipation	Non- constipation	Constipation	Non- constipation	Constipation	Non- constipation	Constipation
Frequency of defecation	25(41.7%)	0(0%)	34(56.7%)	15(25.0%)	0(0%)	16(26.7%)	1(1.6%)	29(48.3%)
Stool characteristics	30(50.0%)	0(0%)	30(50%)	23(38.3%)	0(0%)	15(25.0%)	0(0%)	22(36.7%)
Time of defecation	35(58.3%)	0(0%)	25(41.7%)	23(38.3%)	0(0%)	21(35.0%)	0(0%)	16(26.7%)
Bloating	32(53.3%)	0(0%)	28(46.7%)	10(16.7%)	0(0%)	23(38.3%)	0(0%)	27(45.0%)
Difficulty defecating	30(50.0%)	0(0%)	30(50.0%)	18(30.0%)	0(0%)	17(28.3%)	0(0%)	25(41.7%)
Falling and swelling feeling	23(38.3%)	6(10.0%)	31(51.7%)	17(28.3%)	5(8.3%)	20(33.4%)	1(1.6%)	17(28.3%)

### 3.2 Questionnaires results of the two groups

As shown in **Table 2**, the median values of constipation symptoms in the two groups were 12.00 and 3.00, respectively (p<0.001). The GCSI values of the two groups were 27.00 and 22.00, respectively (p<0.001). The median VSI of the two groups were 25.00 and 22.00, respectively, and the p value was 0.004. The average PAGI-SYM values of the two groups were 52.38 and 44.83, respectively, and the p value was 0.013. The median values of PAGI-QOL of the two groups were 87.00 and 73.00, respectively, with a p value less than 0.001. These results suggest that there are significant differences in the above indicators between the two groups in terms of gastrointestinal symptoms, sensitivity, clinical manifestations and their severity grading.

**Table 2:** Comparison of each scale scores between the diabetic patients with constipation and without constipation.

Assessment Scale	Non-constipation	Constipation	T	p value
Constipation Symptoms	3.00(2.25, 4.00)	12.00(11.00, 13.00)	9.514	<0.001***
GCSI	22.00(20.00, 25.00)	27.00(24.00, 30.00)	5.515	<0.001***
VSI	22.00(20.00, 24.00)	25.00(21.00, 29.75)	2.886	0.004**
PAGI-SYM	44.83 ± 15.93	52.38 ± 9.85	2.55	0.013*
PAGI-QOL	73.00(64.00, 79.00)	87.00(73.00, 94.00)	4.559	*<*0.001***

GCSI, Gastroparesis Cardinal Symptom Index; VSI, Intestinal sensitivity index; PAGI-SYM, Patient Assessment of Upper Gastrointestinal Symptom Severity Index; PAGI-QOL, Patient Assessment of Quality of Life Index. Values are presented as median (first quartile, third quartile) for comparisons using the Mann-Whitney U test, and as mean ± standard deviation for comparisons using Student’s t test. ^***^p<0.001, ^**^p<0.01, ^*^p<0.05.

### 3.3 Clinical parameters of the two groups

As shown in **Table 3**, the final number of subjects for constipation and non-constipation was 60, respectively. In the constipation group, there were 32 males and 28 females, with a total of 12 people aged < 45 years, 22 people aged 45–60 years, and 26 people aged ≥60 years. In the non-constipation group, there were 29 males and 31 females, with a total of 11 persons aged < 45 years, 23 persons aged 45–60 years, and 26 persons aged ≥60 years. In the constipation group, the duration of diabetes was less than 5 years in 9 patients, 5–10 years in 27 patients, and ≥10 years in 24 patients. In the non-constipation group, the duration of diabetes was less than 5 years in 24 patients, 5–10 years in 18 patients and ≥10 years in 18 patients. In the constipation group, 12 patients were thin, 18 patients had normal BMI, 18 patients were overweight and 12 patients were obese. In the non-constipation group, 13 patients were thin, 24 patients had normal BMI, 11 patients were overweight, and 15 patients were obese. The results suggested that there was a significant difference in the duration of diabetes between the two groups (p < 0.01).

**Table 3:** Comparison of differences in clinical parameters between the diabetic patients with constipation and without constipation.

Characteristic	Category	Non-constipation	Constipation	χ²	p value
Gender	Male	29	32	0.300	0.584
	Female	31	28		
Age	<45	11	12	0.066	0.968
	45-60	23	22		
	≥60	26	26		
Course	<5	24	9	9.480	0.009**
	5-10	18	27		
	≥10	18	24		
BMI	Thin	13	12	2.290	0.514
	Normal	21	18		
	Overweight	11	18		
	Obesity	15	12		

BMI, Body Mass Index. ^**^p<0.01.

### 3.4 Chronic diabetic complications of the two groups

As shown in **Table 4**, 38 and 39 patients in the constipation group and the non-constipation group had diabetic retinopathy, respectively. There were 28 and 31 patients with diabetic peripheral neuropathy, 28 and 27 patients with peripheral vascular disease, 32 and 20 patients with diabetic autonomic neuropathy, 28 and 16 patients with diabetic nephropathy in the constipation group and the non-constipation group, respectively (p < 0.05).

**Table 4:** Comparison of differences in diabetic complications between the diabetic patients with constipation and without constipation.

Complications	Non-constipation	Constipation	χ²	p value
DR	39	38	0.190	0.849
	21	22		
DPN	31	28	0.548	0.584
	29	32		
PVD	27	28	0.182	0.855
	33	32		
DAN	20	32	2.211	0.027*
	40	28		
DKD	16	28	2.273	0.023*
	44	32		

DR, Diabetic Retinopathy; DPN, Diabetic Peripheral Neuropathy; PVD, Peripheral Vascular Disease; DAN, Diabetic Autonomic Neuropathy; DKD, Diabetic Kidney Disease; ^*^p<0.05.

### 3.5 Biochemical parameters of the two groups

As shown in **Table 5**, the differences of FBG, 2hPBG, HbA1c, TG, HDL-C, TC, LDL-C, FCP and HOMA-IR between the two groups were compared. The results indicated that HbA1c in the constipation group was significantly higher than that in the non-constipation group, and there was a significant difference between the groups (p < 0.05). TC and LDL-C in the constipation group were significantly higher than those in the non-constipation group, and there were significant differences between the two groups (p < 0.05). The FCP of the constipation group was significantly lower than that of the non-constipation group, and there was a significant difference between the groups (p < 0.05).

**Table 5:** Comparison of biochemical parameters between the diabetic patients with constipation and without constipation.

Parameters	Non-constipation	Constipation	T	p value
FBG	6.91 ± 1.03	6.98 ± 1.15	0.365	0.716
2hPBG	16.91 ± 3.89	16.11 ± 4.57	1.041	0.300
HbA1c	9.37 ± 2.82	10.51 ± 2.09	2.511	0.013*
TG	1.36 ± 0.40	1.39 ± 0.35	0.402	0.688
TC	4.72 ± 1.21	5.50 ± 1.63	2.965	0.004**
HDL-C	1.03 ± 0.20	1.04 ± 0.19	0.315	0.753
LDL-C	3.02 ± 0.76	3.62 ± 0.82	4.175	<0.001***
FCP	1.05 ± 0.33	0.90 ± 0.21	2.911	0.004**
HOMA-IR	0.08(0.07, 0.09)	0.08(0.07, 0.09)	0.016	0.987

FBG, Fasting Blood Glucose; 2hPBG, 2 hours Postgrandial Blood Glucose; HbA1c, Glycosylated Hemoglobin Type A1C; TC, Total Cholesterol; TG, Triglyceride; HDL-C, High Density Lipoprotein Cholesterol; LDL-C, Low Density Lipoprotein Cholesterol; FCP, Fasting C-Peptide; HOMA-IR, Homeostasis Model Assessment, Insulin Resistance; ^***^p < 0.001, ^**^p<0.01, ^*^p<0.05.

### 3.6 TIR and CV values of the two groups

**Figure 1:**
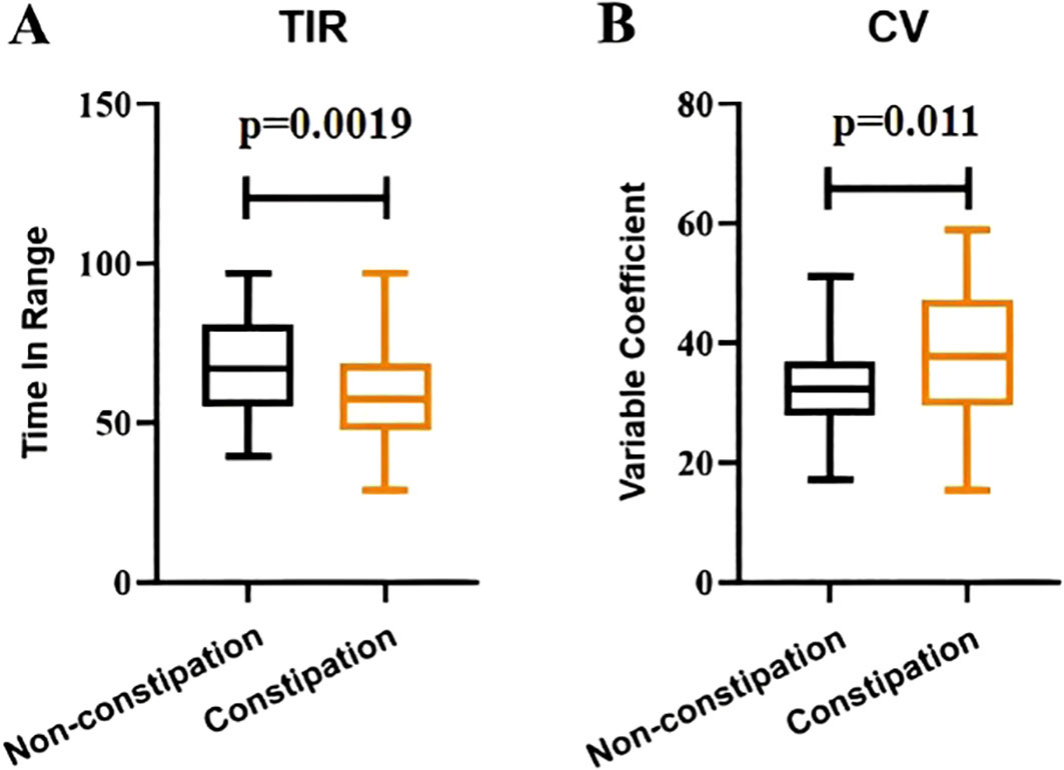
Comparison of TIR and CV between the diabetic patients with constipation and without constipation.

As shown in **Figure 1**, the mean values of TIR in the constipation group and the non-constipation group were 58.84 and 67.97, respectively, showing a significant difference between the two groups (p < 0.05). In addition, the mean values of CV in the constipation group and the non-constipation group were 38.38 and 32.37, respectively, with a significant difference between the two groups (p < 0.05).

### 3.7 Risk factor analysis of constipation in the patients with T2DM

Multivariate Logistic regression analysis was performed, with or without constipation as the dependent variable, and diabetes duration, HbAlc, TC, LDL-C, FCP, TIR and CV as the independent variables. As shown in **Table 6**, TC, LDL-C, TIR and CV were all significantly correlated with constipation (p < 0.05).

**Table 6:** Multivariate logistic regression analysis of the risk of diabetes mellitus with constipation.

Variables	β	OR	95%CI	p value
Course	0.643	1.902	0.926-3.904	0.080
HbA1c	0.128	1.136	0.909-1.421	0.262
TC	0.433	1.542	1.074-2.213	0.019*
LDL-C	0.937	2.551	1.308-4.976	0.006**
FCP	-0.143	0.867	0.608-1.236	0.430
TIR	-0.051	0.950	0.917-0.984	0.005**
CV	0.079	1.082	1.019-1.150	0.011*

HbA1c, Glycosylated Hemoglobin Type A1C; TC, Total Cholesterol; LDL-C, Low Density Lipoprotein Cholesterol; FCP, Fasting C-Peptide; TIR, Time In Range, CV, Coefficient of Variability; ^**^p<0.01; ^*^p<0.05.

### 3.8 TIR and CV ROC curves of the two groups

**Figure 2:**
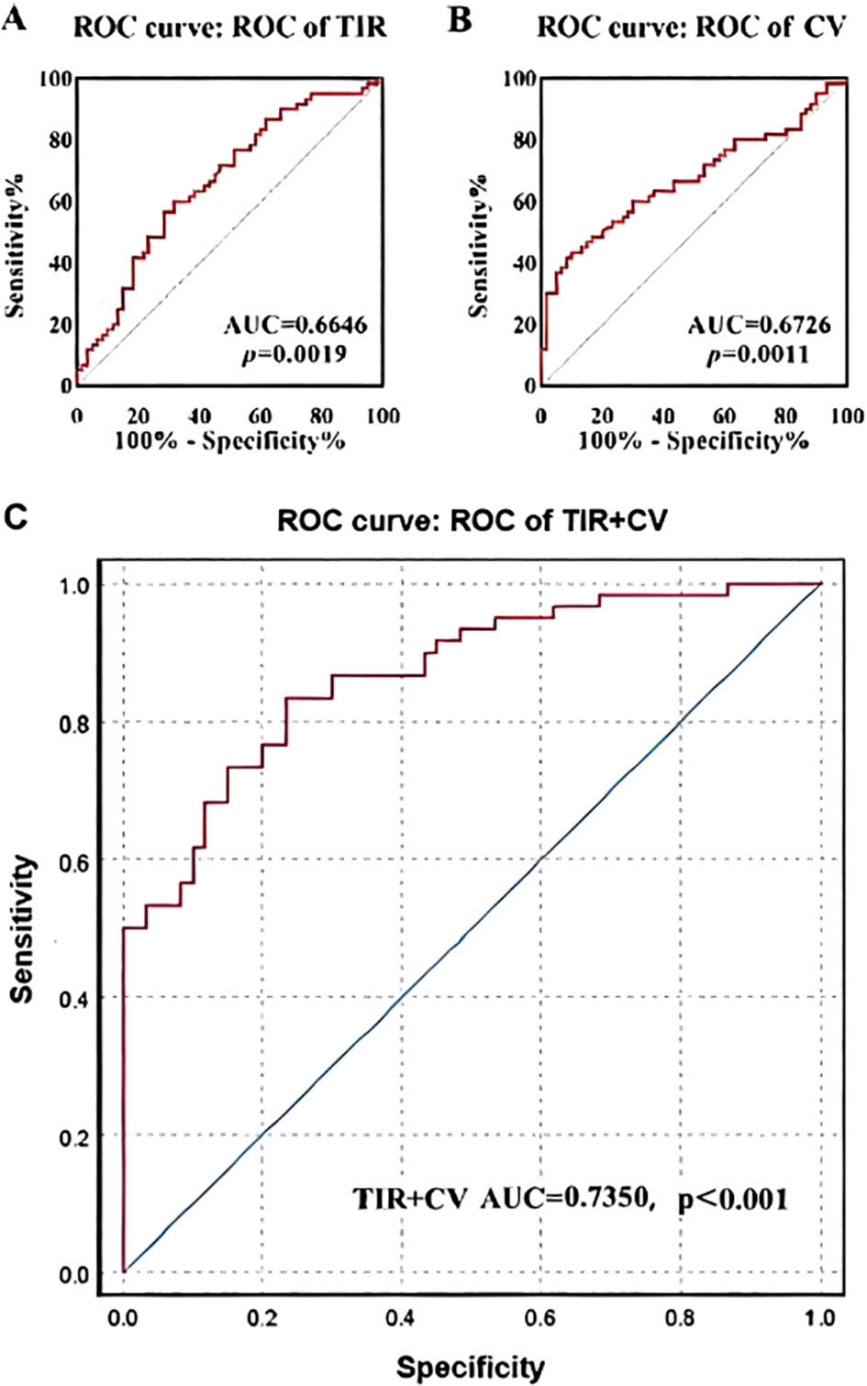
ROC curve analysis of the predictive value of TIR and CV for the risk of diabetes mellitus with constipation.

ROC curve was used to evaluate the effects of TIR and CV on constipation in patients with T2DM. As shown in **Figure 2**, the AUC of the ROC curve analysis of TIR for predicting constipation was 0.6646, with a p value of 0.0019. The AUC of CV in ROC curve analysis for predicting constipation was 0.6726, with a p value of 0.0011. The AUC of TIR+CV combined prediction of constipation in ROC curve analysis was 0.7350 (p<0.001).

## 4 Discussion

Diabetes mellitus is a major global health challenge, with its prevalence and associated complications placing a significant burden on individuals and healthcare systems. Over the past four decades, the number of adults with diabetes in the world has increased four times, and it is estimated that the global patients with diabetes will reach 783.2 million by 2045 (16), and 90-95% of the cases are T2DM. Among diabetic complications, the prevalence of constipation in diabetes is about 25%, seriously affecting the quality of life of diabetic patients, and even resulting in cardiac vascular disease, the perforation of the intestine, and sudden death (17). The definition of constipation in Roman IV Criteria, more than two of the following items are required to occur at least once a week and last for a month: defecate less than twice a week, fecal incontinence more than once a week, be painful when defecating, and the feces are large and even clogging the toilet (18). However, compared to other diabetic complications such as retinopathy or nephropathy, the associated factors and pathophysiology of diabetic constipation remain less well-characterized. This study aims to address this gap by exploring the clinical characteristics and related factors of patients with T2DM complicated with constipation, especially the potential association between the new CGM - derived indicators - TIR and CV - and constipation in patients with T2DM.

In this cross-sectional study, the course of the patients in constipation group was significantly higher than that of non-constipation, which suggested that the longer time of the disease, the greater the risk of constipation, which was consistent with the previous study (19). Although the significance of the course in the multivariate regression analysis is weakened, it may be related to the small sample sizes and selective bias.

Among several measures of islet function, C-peptide, which accompanies insulin secretion at equal molar concentration, has been shown to be a more reliable indicator of islet β-cell function. C-peptide measurements are commonly used as endpoints in clinical trials aimed at preventing diabetes and further progression of diabetes (20). In addition, C-peptide is closely related to chronic complications of diabetes, FCP is a protective factor for diabetic retinopathy, and the incidence of diabetic retinopathy decreased with the increase of FCP level (21). Although the decreased FCP values were also found to be more pronounced in the constipation group in the present study, it was not significant in the multivariate regression analysis, suggesting that decreased FCP is not an important associated factor for diabetes complicated with constipation.

Normal lipid metabolism, such as lipid uptake, synthesis, and hydrolysis, is essential for maintaining cellular homeostasis. Glycolipids and phospholipids together with cholesterol constitute the main components of biofilms. Cholesterol is also a substrate for the synthesis of fat-soluble vitamins and steroid hormones (22). TC and LDL-C are commonly used to reflect lipid levels. The present study found that TC and LDL-C were significantly increased in patients with constipation compared to the non-constipated group, suggesting that patients with constipation had higher lipid levels. A similar study reported that hyperlipidemia is a risk factor for chronic constipation (23).Blood glucose level is one of the most important risk factors for chronic complications of diabetes. HbA1c levels was usually used to assess the glycemic control conditions. Impaired glycemic control as assessed by HbA1c is a strong predictor of diabetes onset and diabetic complications. The results of the present study showed that the level of HbA1c in the constipation group was higher than that in the non-constipation group, although it did not show significance in the regression analysis, which might be related to the selective bias, because the included patients in the present study were all hospitalized patients with hyperglycemia, and the difference in HbA1c was not obvious.

In addition to HbA1c, glucose variability explains glucose fluctuations and has become a commonly used assessment tool with advances in continuous glucose monitoring (CGM) technology (24). TIR represents the proportion of time that the blood glucose level is within the target range (25). A recent review have shown a very strong relationship between TIR and chronic complications of diabetes (26). It was reported that TIR was associated with the degree of painful neuropathy in diabetic patients, independent of HbA1c level, other blood glucose variability indicators and risk factors, and is a valuable clinical evaluation index for diabetic patients (27). Another study found that the prevalence of diabetic retinopathy based on severity decreased as the TIR quartile increased, and the severity of diabetic retinopathy was negatively correlated with the TIR quartile (28). It was also reported that TIR is a protective factor for the risk of diabetic nephropathy in T2DM patients, and lower TIR can increase the risk of diabetic nephropathy (29). In the present study, TIR was significantly lower in the constipation group than in the non-constipation group, and low level of TIR was an associated factor for constipation in T2DM patients.

CV is the most appropriate measure to assess the daily mean blood glucose variability. A CV of 33% was set as the glucose variability threshold for Chinese patients with diabetes. One study showed that elevated CV was a risk factor for the occurrence of peripheral neuropathy in T2DM, which had a good predictive value for the occurrence of DPN (30). In addition, in a related study of diabetes complicated with chronic kidney disease, CV of blood glucose is an independent risk factor for increased ESRD (31). The present study found that CV increased significantly in the constipation group compared with the non-constipation group, suggesting a significant correlation between CV and constipation, and higher CV increased the risk of constipation in T2DM patients.

In related studies of diabetes and its associated complications, TIR and CV are often jointly tested. A study found that TIR and CV was associated with the presence of composite microvascular complications (32). In addition, it was also found that TIR and CV were significantly correlated with changes in the tissue characteristics of carotid artery wall (33). Our cross-sectional analysis identified significant associations between both lower TIR and higher CV and the presence of constipation. These findings align with and extend the growing body of literature that links suboptimal glycemic control, especially glucose variability, to various diabetic complications. The observed associations are biologically plausible. Chronic hyperglycemia and acute glycemic fluctuations may contribute to autonomic neuropathy, a well-known mechanism in diabetic enteropathy, which can impair colonic motility and transit. Thus, TIR and CV, as integrated measures of glycemic exposure and stability, may reflect aspects of metabolic dysregulation relevant to gastrointestinal dysfunction.

It is crucial to emphasize that our findings are hypothesis-generating and do not establish causality. The cross-sectional nature of our study means we cannot determine the direction of the observed associations. Alternative explanations must be considered. First, constipation itself, through alterations in diet, gut microbiota, or medication use such as certain laxatives or opioids, might adversely affect glycemic stability. Second, unmeasured confounding is a significant consideration. Notably, we lacked detailed data on medications known to influence both glucose metabolism and gastrointestinal function, such as GLP-1 receptor agonists and metformin. Their use could confound the relationship between CGM metrics and constipation. Therefore, TIR and CV might more appropriately be viewed as potential markers or indicators of a pathophysiological state that predisposes to both poor glycemic control and constipation, rather than as direct causative factors.

Our analysis also examined traditional risk factors. While longer diabetes duration and higher levels of TC and LDL-C showed univariate associations with constipation, their significance was attenuated in the multivariate model that included TIR and CV. This suggests that the information captured by these dynamic CGM metrics may overlap with or be more directly relevant to constipation than some conventional static measures. The lack of a significant independent association for FCP and HbA1c in our final model may be related to sample characteristics or the possibility that TIR and CV capture distinct, and perhaps more proximate, aspects of dysglycemia relevant to enteric neuropathy.

Our study has several important limitations that must be acknowledged when interpreting the results. The cross-sectional design precludes any inference of causality or temporal sequence between glycemic control and constipation. In addition, the use of a single-center, hospitalized patient cohort may limit the generalizability of our findings to the broader, community-dwelling T2DM population. And then, we lacked comprehensive data on medications (especially GLP-1 agonists and metformin), dietary fiber intake, physical activity, and specific laxative use, all of which are potential confounders. Moreover, constipation was defined using the Rome IV criteria based on patient-reported symptoms. The absence of objective measures of colonic transit such as radiopaque marker studies is a limitation. Exposure Measurement: The CGM monitoring period was limited. Longer monitoring might provide a more robust representation of habitual glycemic variability. The assessment accuracy of the models, as indicated by AUC values ranging from 0.66 to 0.73, is modest. This underscores that TIR and CV are not standalone diagnostic tools but may contribute to risk stratification. As noted, we cannot determine whether poorer glycemic control (reflected by lower TIR/higher CV) contributes to the development of constipation or vice versa.

To address the limitations of this study and build upon its findings, longitudinal studies are needed to establish the temporal relationship between TIR/CV and the incidence of new-onset constipation, which would strengthen evidence for a potential causal link. Future analyses should rigorously account for the effects of glucose-lowering and gastrointestinal medications. Furthermore, interventional trials are warranted to investigate whether therapies aimed at improving TIR and reducing CV can prevent or alleviate constipation in T2DM. Research integrating CGM with objective assessments of autonomic neuropathy such as cardiovascular reflex tests and enteric nerve function is essential to elucidate the biological pathways connecting glucose variability to colonic dysmotility.

## 5 Conclusion

Lower TIR and higher CV were associated with constipation in T2DM patients. These findings suggest that CGM-derived metrics may be useful markers of constipation risk and warrant investigation in prospective studies to determine whether improving these parameters can prevent constipation development.

## Data Availability

The raw data supporting the conclusions of this article will be made available by the authors, without undue reservation.
